# The effects of temperament type on infusion extravasation in newborns

**DOI:** 10.1038/s41598-024-66218-3

**Published:** 2024-07-04

**Authors:** Fang Huang, Li-xuan Huang, Zhen-peng Huang, Jiao-jiao Wei, Chang-jiang Lu

**Affiliations:** 1 Department of Neonatology, Nanning Maternty and Child Health Hospital, Nanning, China; 2 Outpatient Department, Nanning Maternity and Child Health Hospital, Nanning, China; 3 Faculty of Nursing, Guangxi University of Chinese Medicine, Guangxi, China; 4 Department of Otolaryngology and Head-Neck Surgery, Nanning Maternity and Child Health Hospital, Nanning, China; 5 Nursing Department, Nanning Maternity and Child Health Hospital, Nanning, China

**Keywords:** Newborn, Infusion extravasation, Temperament, The neonatal behavioral assessment scale, Psychology, Health care, Medical research

## Abstract

Infusion extravasation has an increased incidence in newborns, which can result in various adverse outcomes. This study aimed to investigate the effects of different types of temperament on infusion extravasation in newborns. A total of 209 newborns aged 4–7 days who were treated with infusion therapy were assessed for temperament type using the neonatal behavioral assessment scale score (NBAS). The 2009 Infusion Nurses Society clinical grading criteria for extravasation were used, and the clinical data of the newborns, such as gestational age and body weight, were collected. Out of 209 newborns assessed, 107 developed infusion extravasations, with an incidence rate of 51.2%. Newborns with intermediate temperament type were more prone to develop infusion extravasation. Newborns with low body weight, amniotic fluid aspiration syndrome, or meconium aspiration syndrome were prone to develop infusion extravasation. Body weight, temperament type of consolability, temperament type of peak of excitement, diseases, general temperament type, and NBAS total scores of the neonates were independent risk factors for infusion extravasation. Thus, different types of temperament can have an impact on neonatal extravasation.

## Introduction

Extravasation is the accidental leakage of an intravenously administered solution into the surrounding tissues, which is a common complication of intravenous infusion therapy that can cause serious injury without timely treatment^1,2^. Infusion extravasation occurs in up to 70% of newborns treated in the neonatal intensive care unit (NICU), and approximately 4% of the affected newborns leave the NICU with a scar due to infusion extravasation^3,4^. Furthermore, severe infusion extravasation can lead to scar formation, which could be additionally accompanied by malformation and functional damage, and even secondary infection that could potentially be fatal. Infusion extravasation causes serious damage to newborns, which prolongs the hospitalization and nursing time and increases treatment costs^5,6^.

Recent studies have suggested that infusion extravasation could be affected by factors related to the newborns, caregivers, medications, indwelling needles, and diseases^7–9^. Moreover, most hospitalized newborns are treated with total parenteral nutrition and calcium due to diseases, and these infusions could be hypertonic, hypotonic, vasoconstrictor, or other irritant solutions that can increase the incidence of infusion extravasation^10^. Furthermore, since neonatal veins are small and fragile, the selection and application of infusion tools and devices also affect the occurrence of infusion extravasation^11^.

Temperament is a relatively stable biologically based inborn behavioral tendency of infants exhibited early after birth, which is the external manifestation of infant behaviors^12^. It affects not only the interaction between children and the environment but also their exploration of the external environment and learning knowledge, which plays an important role in their social development^13^. Different temperament types have specific effects on the occurrence of diseases, and the classification and determination of temperament type are helpful for the early detection of diseases^14,15^. However, the influence of different newborn temperament types on the occurrence of infusion extravasation remains unknown. Premature and sick neonates are more likely to have extravasation compared with mature and healthy neonates^3–6^. Recent studies have suggested that the prevention of extravasation in newborns should be emphasized, but only a few studies have focused on the prevention of extravasation caused by factors related to newborns^16^.

The neonatal behavioral assessment scale (NBAS), developed in the year 1973 and revised in the year 1995 by Brazelton TB, is used to assess a newborn’s health, maturity, and temperament during the first four weeks of life, and can be regarded as a tool for assessing temperament early in the first month of life^17–20^. The NBAS is used to analyze and classify neonatal temperament into three types according to the average scores of items, namely excitatory, mild, and intermediate types, which are equivalent to the active, quiet, and intermediate types classified by Brazelton TB. Multiple recent studies have confirmed that both scales are consistent with each other^21,22^.

This study aimed to investigate the effects of different temperament types and neonatal temperament factors on the occurrence of infusion extravasation in order to increase the clinical and conceptual knowledge to help prevent its development in newborns.

## Materials and methods

### Participants

This study was a prospective cross-sectional study, and the sample size was calculated using the formula N = Z^2^ 1−α/2(1−*p*)/ε^2^ *p*^23^. Previous studies have reported that the incidence of infusion extravasation in newborns is 70%^24^. According to the formula, the minimum sample size for this study was 192 participants.

This study enrolled 209 hospitalized newborns, including 147 males and 62 females, aged 4–7 days (5.04 ± 1.05 days), with gestational ages ranging from 37 to 42 weeks (38.99 ± 1.27 weeks) and body weights ranging from 2000 to 4380 g (3041.24 ± 462.89 g), without congenital malformations and without maternal complications during pregnancy from January to September 2022. All newborns underwent brain ultrasound, brainstem auditory evoked potentials, electroencephalogram, blood calcium, phosphorus, alkaline phosphatase, and hemoglobin examinations within three days of birth to exclude the influence of hidden diseases. Peripheral venous catheters were inserted during hospitalization in the NICU, and newborns with successful placement at the first time were evaluated.

Written informed consents were obtained from all newborn patients’ parents or their family members. This study and written informed consents were approved by the Institutional Ethical Committee of Nanning Maternity and Child Health Hospital. All methods were carried out in accordance with relevant guidelines and regulations according to the Declaration of Helsinki.

### Study measurement

#### Neonatal behavioral neurological assessment

We used the neonatal behavioral assessment scale (NBAS) to assess the temperament types of the newborns, and the Chinese version scale sensitivity is 88.9% and specificity is 82.5%^17,25^. The different temperament types of the newborns were preliminarily assessed based on the scores of six behavioral items related to the neonatal temperament classification, including consolability with intervention, peak of excitement, rapidity of buildup, irritability, lability of states, and self-quieting activity. For consolability with intervention item scores, 1–3 points meant high level, 4–6 points meant medium level, and 7–9 points meant low level; except for that, all other items scores are divided into low level for 1–3 points, medium level for 4–6 points, and high levels for 7–9 points based on the average score of the 9-point scale^26,27^. The different temperament types were then divided into three groups according to scores from 1 to 9 of each behavioral item: excitatory, intermediate, and mild. The three temperament types, namely mild, intermediate, and excitatory, were determined according to the total scores from 1 to 54 of the assessment, with 6–18 points classified as mild, 19–36 points as intermediate type, and 37–54 points as excitatory type^26,27^.

Newborns aged 4–7 days were assessed by a trained and qualified examinator at the interval between feedings and the beginning of sleep in a quiet, semi-dark environment at a room temperature of 24–28 ℃. The assessment items and the duration of each assessment were recorded, and all assessments were completed within 10 min^27^.

#### Clinical grading criteria for extravasation

In this study, the 2009 Infusion Nurses Society clinical grading criteria for extravasation were used to evaluate the degree of infusion extravasation in newborns. The grading of infusion extravasation was as follows: grade 0, no relevant symptoms; grade 1, blanched skin and edema with a maximum diameter less than 2.5 cm, with or without pain; grade 2, blanched skin and edema with a maximum diameter from 2.5 to 15 cm, cool to touch, with or without pain; grade 3, blanched skin, translucent, edema with a maximum diameter over 15 cm, cool to touch, mild to moderate pain, possible numbness; grade 4, blanched skin, translucent, tight skin, leaking, discolored skin, bruised, swollen, edema with a minimum diameter over 15 cm, deep pitting tissue edema, circulatory impairment, moderate to severe pain, blood product, irritant, or vesicant infiltration^28^.

Once infusion extravasation was detected, the evaluator immediately measured the maximum diameter of the extravasation site of the newborn using a ruler, measured the skin temperature with a clinical thermometer, and judged the severity of neonatal infusion extravasation according to the grading criteria.

Clinical data, such as gestational age, body weight, and diseases of all newborns, were collected from the hospital information system.

### Statistical analysis

Statistical analysis was conducted using IBM SPSS Statistics version 26.0. Continuous variables were expressed as mean ± standard deviation (*x* ± *s*). Categorical variables were represented as frequencies and percentages. Student's t-test was used to analyze the comparison of continuous variables. Comparison of categorical variables was performed using the chi-square test or χ^2^ test. Multivariable logistic regression analysis was employed to identify independent factors influencing extravasation of intravenous fluids in newborns. Statistical significance was set at *P* < 0.05.

### Ethical approval

Written informed consents were obtained from all newborn patients’ parents or their family members. The study and verbal informed consents were approved by the Institutional Ethical Committee of Nanning Maternity and Child Health Hospital. All study methods were carried out in accordance with relevant guidelines and regulations according to the Declaration of Helsinki.

## Results

### Occurrence and clinical feature on neonatal infusion extravasation incidence

In this study, a total of 107 newborns developed infusion extravasation, accounting for 51.2%, indicating a relatively high incidence of extravasation in newborns. Among the 107 newborns with infusion extravasation, newborns with low body weights were more prone to infusion extravasation (*t* = − 5.83, *P* < 0.05) (Table 1). In addition, the incidence of infusion extravasation was higher with amniotic fluid aspiration syndrome and meconium aspiration syndrome (*x*^2^ = 33.48, *P* < 0.05) (Table 2).

**Table 1:** Clinical feature and neonatal infusion extravasation.

		Infusion extravasation	Without infusion extravasation	
Gender				
	Male	80 (74.77%)	67 (65.69%)	
	Female	27 (25.23%)	35 (34.31%)	*v* = 1, *x*^2^ = 2.06, *P* = 0.15
Age (d)		5.00 ± 1.08	5.07 ± 1.02	*df* = 207, *t* = 0.47, *P* = 0.64
Gestational Ages (d)		39.23 ± 1.15	38.71 ± 1.32	*df* = 207, *t* = − 3.31, *P* = 0.64
Weight (g)		3210.79 ± 414.27	2863.38 ± 445.82	*df* = 207, *t* = − 5.83, *P* < 0.01

**Table 2:** Diseases and neonatal infusion extravasation [n (%)] *.

	Amniotic fluid aspiration syndrome	Neonatal pneumonia	Neonatal hyperbilirubinemia	Meconium aspiration syndrome	Other diseases
Infusion Extravasation	39 (36.45)	15 (14.02)	12 (11.21)	11 (10.28)	30 (28.04)
Without Infusion Extravasation	11 (10.78)	17 (16.67)	42 (41.18)	1 (0.98)	31 (30.39)

* *df* = 4, *x*^2^ = 33.48, *P* = 0.00.

### Role of neonatal behaviors and temperament types on infusion extravasation

In this study, good consolability and the total score of temperament assessment were significantly correlated with the incidence of infusion extravasation in newborns (*t* value were 12.85 and 7.15, respectively; all *P* < 0.05) (Table 3). Among the newborns with infusion extravasation, the dominant temperament type was the intermediate type (*x*^2^ = 13.68, *P* < 0.05). The primary behaviors that influenced infusion extravasation were consolability and peak of excitement (*x*^2^ value were 96.95 and 38.43, respectively; both *P* < 0.05) (Table 4).

**Table 3:** NBNA assessment and neonatal infusion extravasation (*x* ± *s*).

	Infusion extravasation	Without infusion extravasation	
Consolability with Intervention	8.64 ± 1.08	5.90 ± 1.88	*df* = 207, *t* = 12.85, *P* < 0.01
Peak of Excitement	2.49 ± 0.86	3.37 ± 0.97	*df* = 207, *t* = 0.47, *P* = 0.64
Rapidity of Buildup	1.19 ± 1.12	1.07 ± 0.77	*df* = 207, *t* = 0.83, *P* = 0.41
Irritability	1.17 ± 0.37	1.22 ± 0.98	*df* = 207, *t* = -0.47, *P* = 0.64
Lability of States	1.27 ± 0.47	1.22 ± 0.98	*df* = 207, *t* = 0.56, *P* = 0.57
Self-quieting Activity	8.65 ± 0.77	8.57 ± 0.86	*df* = 207, *t* = 0.68, *P* = 0.50
Total Scores of Temperaments	23.28 ± 1.64	21.46 ± 3.44	*df* = 207, *t* = 7.15, *P* < 0.01

**Table 4:** Temperaments type and neonatal infusion extravasation [n (%)].

Infusion extravasation	Temperaments type	Consolability with intervention *	Peak of excitement **	Rapidity of buildup	Irritability	Lability of states	Self-quieting activity	Temperaments type ***
Yes								
	Mild	23 (21.50)	35 (32.71)	106 (99.07)	105 (98.13)	105 (98.13)	105 (98.13)	14 (13.08)
	Intermediate	83 (77.57)	72 (67.29)	0 (0)	0 (0)	2 (1.87)	2 (1.87)	91 (85.05)
	Excitatory	1 (0.93)	0 (0)	1 (0.93)	2 (1.87)	0 (0)	0 (0)	2 (1.87)
No								
	Mild	91 (89.22)	77 (75.49)	100 (98.04)	102 (100)	102 (100)	102 (100)	1 (0.98)
	Intermediate	11 (10.78)	25 (24.51)	0 (0)	0 (0)	0 (0)	0 (0)	101 (99.02)
	Excitatory	0 (0)	0 (0)	2 (1.96)	0 (0)	0 (0)	0 (0)	0 (0)

* *df* = 2, *x*^2^ = 96.65, *P* < 0.01.

** *df* = 2, *x*^2^ = 38.43, *P* < 0.01.

*** *df* = 2, *x*^2^ = 13.68, *P* < 0.01.

### Logistic regression analysis of independent factors for neonatal infusion extravasation

The findings of this study showed that body weight (OR = 3.45, 95%C.I. 1.58–7.50), diseases (OR = 1.24, 95%C.I. 1.08–1.43), temperament type of consolability (OR = 0.04, 95%C.I. 0.02–0.10), and temperaments type (OR = 0.28, 95%C.I. 0.19–0.41) were independent factors for infusion extravasation incidence in newborns (all *P* < 0.05) (Table 5).

**Table 5:** Logistic regression analysis for neonatal infusion extravasation.

	B	S.E	Wald	Sig	Exp (B)	95%C.I
Weight	1.24	0.40	9.74	< 0.01	3.45	(1.58, 7.50)
Disease	0.21	0.07	8.90	< 0.01	1.24	(1.08, 1.43)
Consolability with Intervention	–3.14	0.44	51.87	< 0.01	0.04	(0.02, 0.10)
Peak of Excitement	–0.97	0.41	5.53	0.02	0.38	(0.17, 0.85)
Temperaments Type	–1.28	0.20	39.76	< 0.01	0.28	(0.19, 0.41)

## Discussion

For newborns, infusion extravasation is not only accompanied by pain but also increases the risk of infection due to impaired skin integrity and can cause disability and death due to its escalation^3,5^. Furthermore, the adverse consequences caused by infusion extravasation often require more therapeutic drugs and care, increasing treatment costs and social and economic burdens^29^.

Some neonatal diseases such as amniotic fluid aspiration and meconium aspiration, would affect vascular or subcutaneous lesions, and then resulting in infusion extravasation^30,31^. In this study, we have found out that amniotic fluid aspiration syndrome and meconium aspiration syndrome would have an impact on infusion extravasation in newborns. Inhalation of amniotic fluid during delivery by a fetus result in its rapid absorption by the alveolar capillaries. The sebum and exfoliated keratinized epithelial cells in the amniotic fluid can result in chemical and mechanical stimulation of the alveoli, increasing the permeability of the pulmonary blood vessels and decreasing gas diffusion, causing alveolar edema and even collapse. Meanwhile, due to the decrease in pH value and PaO_2_ and the increase in PaCO_2_, the newborn is in a hypercoagulable state, which leads to hypoxemia, hypercapnia, and severe vasoconstriction, increasing the difficulty in placing the peripheral indwelling needle and the incidence of infusion extravasation^30,31^. Moreover, when neonatal meconium aspiration syndrome occurs, the amniotic fluid contaminated by feces is inhaled by the fetus into the respiratory tract, causing mechanical obstruction and chemical inflammation of the alveoli and respiratory tract. Since the meconium-contaminated amniotic fluid contains many substances, such as bile acid, large particles of meconium, and bilirubin, their mixture results in high viscosity that can easily block the small trachea and bronchi of the newborns, causing neonatal hypercapnia and hypoxemia, leading to vasoconstriction, which further causes increased peripheral vascular resistance, increasing the difficulty of venipuncture and inducing infusion extravasation^32,33^.

Temperament refers to individual differences that are biologically based. It is a component of personality, which may change over time, and reflects behavioral tendencies; besides, temperament is inborn, from birth, each newborn responds to external stimulations in a unique way, and each newborn has a unique style ^34^. Studies have shown that newborn temperament types are mainly classified into excitatory, mild, and intermediate types, and newborns with different temperament types have different performances^35^.

Temperament, a stable psychological trait to measure individual differences in behavior, can influence and predict individual behaviors to a certain extent. Moreover, children with different temperament types exhibit different behaviors when exposed to the same external stimulation^36^. A previous study that used NBAS to evaluate newborns found that a low level of self-regulation in newborns and infants was correlated with a low level of self-regulation and changes in sleep rhythm^37^. In addition to external factors, neonatal extravasation is also affected by newborn factors, such as vascular elasticity, puncture site, and disease state^37^. This study has found that neonatal extravasation is also affected by temperament type, and newborns with different temperament types had different incidences of infusion extravasation.

Newborns have a low tolerance for pain caused by venipuncture, and are prone to crying and struggling and are discomfort during venipuncture^38^. Excitatory newborns are active during infusion therapy, making it difficult to accurately place the needle and catheter. However, the crying, restlessness, and irritability of newborns results in increased focus from nurses, leading to a reduced occurrence of infusion extravasation. The mild newborns, unlike the ones with excitatory and intermediate temperament types, are quiet, inactive, and more cooperative with the puncture procedure. Even if there is pain caused by infusion extravasation, they rarely show it through crying, restlessness, or other ways, it can only be detected by the nursing rounds in infusion extravasation. For newborns with intermediate temperament type, venipuncture is a very unpleasant stimulation, and they may show irritability and cry but are easy to comfort compared to excitatory newborns. However, they are relatively active during most of their waking hours, and thus they are most likely to experience infusion patch loosening and falling off and indwelling cannula slipping out of the vessel during infusion therapy, with the highest incidence of infusion extravasation^39^.

Among limitations in this study have the following. First, this study was a single-center study. In addition, this study was a cross-sectional open study, and no blinding was used.

In conclusion, the different temperament types of newborns showed to affect neonatal extravasation, suggesting that nurses and healthcare professionals in the neonatal unit should pay attention to newborn temperament too, when conducting intravenous infusion therapy, nursing rounds, and management to prevent the occurrence of infusion extravasation.

## Data Availability

The datasets generated during and/or analyzed during the current study are available from the corresponding author on reasonable request.
